# Improved Fractal Space Filling Curves Hybrid Optimization Algorithm for Vehicle Routing Problem

**DOI:** 10.1155/2015/375163

**Published:** 2015-06-16

**Authors:** Yi-xiang Yue, Tong Zhang, Qun-xing Yue

**Affiliations:** ^1^School of Traffic and Transportation, Beijing Jiaotong University, Beijing 100044, China; ^2^Research Center for Solid Mechanics, Beihang University, Beijing 100191, China; ^3^China Academy of Space Technology (CAST), Beijing 100094, China

## Abstract

Vehicle Routing Problem (VRP) is one of the key issues in optimization of modern logistics system. In this paper, a modified VRP model with hard time window is established and a Hybrid Optimization Algorithm (HOA) based on Fractal Space Filling Curves (SFC) method and Genetic Algorithm (GA) is introduced. By incorporating the proposed algorithm, SFC method can find an initial and feasible solution very fast; GA is used to improve the initial solution. Thereafter, experimental software was developed and a large number of experimental computations from Solomon's benchmark have been studied. The experimental results demonstrate the feasibility and effectiveness of the HOA.

## 1. Introduction

Vehicle Routing Problem (VRP) is an important problem in Supply Chain Management (SCM). The classical Vehicle Routing Problem can be defined as the determination of an optimal set of routes for a fleet of vehicles which need to serve a set of customers. With the time window constraints, general VRP can be transformed to one of the variants of the VRP, the Vehicle Routing Problem with time window (VRPTW). VRPTW has been proved to be an NP-Hard problem and can hardly be found as the global optimal solution [1]. Although VRP has been studied for decades and there are a huge number of heuristic methods to solve the Vehicle Routing Problem to near-optimal solution [2], it is still an important research direction to researchers in SCM. Recently, research attention for VRP has turned to hybridization of metaheuristics. It is assumed that combining features of different heuristics in complementary fashion can result in more robust and effective optimization tools.

Classic Space Filling Curves (SFC), originally described by the Italian mathematician G. Peano in 1890, is based on the fractal theory. The space was divided into adjacent Sierpinski triangles by fractal method until each point in the space can be expressed in a continuous line (0, 1) by space filling curves. The space filling curve represents multidimensional space with one-dimensional space line. The applications of fractal theory have played an important role in image processing and multidimensional data index and so forth.

The locations of all customers and depots can be regarded as the points in the two-dimensional space, and SFC can interpret the sequence of these points; once the sequence of the points is determined, the routes for VRP also can be found. Bartholdi et al. [3, 4] construct a short tour through points in the plane and the points are sequenced as they appear along a space filling curve. Bartholdi's SFCs are constructed in triangle and rectangle and the heuristic consists essentially of sorting. The SFC method is potentially very useful, for it is the fastest available heuristic for large problems. Storer and Bringhurst have tested problems of 10 to 500 cities [5]. But the average solution quality of the method is only fair, and its worst-case performance is relatively bad [6]. So although it is easily coded and requires only *O*(*N*) memory and *O*(*N*log⁡*N*) operations, no papers imply that this method has been applied in some real-world instances.

Genetic Algorithms (GA) are a family of heuristic search procedures based on the biological paradigm of natural selection. They were pioneered by De Jong [7] to solve nonlinear optimization problems and later extended by various authors to many combinatorial problems. A more thorough description of past GA applications to VRP is given in [8–11].

This paper proposes a brand new Hybrid Optimization Algorithm (HOA) combined with SFC and GA. We use SFC to get a feasible original solution and then use GA to obtain the optimal solution. This paper is organized as follows. We provide a more detailed description of VRP and propose a mathematic model in the following section. In Section 3, we introduce the HOA in detail. Subsequently, we use Solomon benchmark to test the HOA and the computational results are analyzed in Section 4. Finally, some conclusions and recommendations for further research are proposed.

## 2. Description and Formulation of VRP

The VRP can be described as “the provision of goods and services from supply points to demand points”; for classic VRP, we consider one supply point and many demand points. The supply point can be a depot and demand points can be customers to ordinary logistic system. We now present a mathematical formulation of the VRPTW problem.

Suppose there are *L* customers and one depot; the depot has the same vehicles with the capacity of *q* tons for each trip. The solution of the problem is to get an order of *L* points and then divide the *L* points into *m* groups; each group means one trip of a vehicle. The goal of VRP is to minimize the total route length and vehicle number.

### 2.1. Presumption

Note that our formulation of VRP has some presumptions as follows:

1. Each trip of the vehicles must start from the depot and end with the depot.
2. The demand volume of each customer should not exceed the vehicle loading capacity.
3. Each customer must be visited only once.
4. Each customer has a service time window.

### 2.2. Formulation

In considering the constraints above, a multiobjective mathematical model is constructed as follows.

The decision variables are

(1) yki=1,if demand point  i  is  visited by vehicle  k0,otherwise,xijk=1,if vehicle  k  traveled from demand point  i  to  j0,otherwise.

The other notations used in this model are defined as follows.

*v* is the average speed of vehicle, *g*_*i*_ is the demand volume of customer *i*, *d*_*ij*_ is the distance from *i* to *j*, *t*_*ij*_ is the travel time from *i* to *j*, *s*_*i*_ is the service time of customer *i*, *R*_*k*_ is the route of vehicle *k* which is also a set of visiting points, and *R*_*k*_ = {*r*_*k*0_, *r*_*k*1_,…, *r*_*kℓ*_,…, *r*_*k*0_}, where *r*_*k*0_ = *r*_0_, ∀*k*, is the depot. *R*_*k*_1__∩*R*_*k*_2__ = *ϕ*, ∀*k*_1_, *k*_2_ ∈ [1, *m*], *k*_1_ ≠ *k*_2_, [*t*_*i*_^*s*^, *t*_*i*_^*e*^] is the time window of customer *i*, and *τ*_*ki*_ is the moment when vehicle *k* arrives at point *i*; for each point *i*, it should obey the constraints *t*_*i*_^*s*^ ≤ *τ*_*ki*_ ≤ *t*_*i*_^*e*^.

The model of VRP can be formulated as follows:

(2) min⁡Z1=∑i=1L ∑j=1L ∑k=1mdij·xijk,min⁡Z2=m

subject to

(3) ∑iLgiykiq∀k∈1,m

(4) ∑k=1myki1∀i∈1,L,  ∀k∈1,m

(5) ∑i=1Lxijkykj∀j∈1,L,  ∀k∈1,m

(6) ∑i=1Lxijkyki∀i∈1,L,  ∀k∈1,m

(7) tjs∑k=1m ∑i=1,i≠jLxijk·ykiτki+si+tij≤tje∀j∈1,L.

Formula (2) represents object functions; the first object is to minimize the total route length, and the second object is to minimize the vehicle number.

Formulae (3)–(7) are constraints. Formula (3) guarantees that the total customers' requirement cannot exceed the vehicle loading capacity. Formula (4) guarantees that each demand point can be visited by one vehicle. Formulae (5) and (6) together mean that each demand point must be visited at least and at most once. Formula (7) means that the arrival time of each requirement point vehicle must meet the service time window of each customer. *τ*_*ki*_ can be calculated as follows:

(8) τki=max⁡τk,i−1+sk,i−1+trk,i−1,rki,tis,where  tij=dijv.

## 3. Hybrid Optimization Algorithm

### 3.1. Outline of the Algorithm

**Figure 1:**
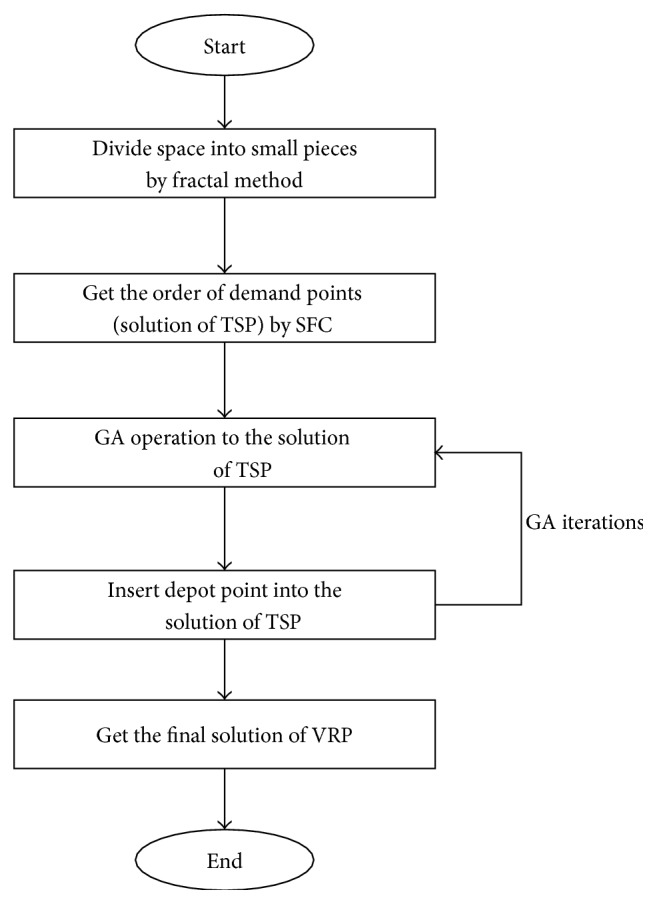
Outline of the algorithm.

When there are no constraints of vehicle loading capacity and time window, VRP can be considered as a Traveling Salesman Problem (TSP), which is to find a sequence of all demand points to get the shortest route distance. So once we get the order of demand points, we can insert several depot points into the queue of demand points according to the constraints of time window and loading capacity. Thus, the solution of the TSP problem can be divided by point of depot into several small fragments; each fragment represents a route for one vehicle which starts from the depot and ends with the depot. The outline of the algorithm is shown in Figure 1.

### 3.2. Process of Algorithm

#### 3.2.1. Honeycomb Based SFC

There are many kinds of space filling curves, and the widely used are Sierpinski SFC and Hilbert SFC. Sierpinski SFC is based on the Sierpinski triangle subdivision, and the Hilbert SFC is based on rectangular segmentation. Honeycomb structure is a new fractal hexagonal lattice structure which can cover any two-dimensional space with seamless lattice [12, 13]. The geometry of honeycomb structure has been extensively studied and has been proven to have high mechanical stability and high thermal efficiency and covering efficiency, which has been applied in many fields, such as nanostructure materials and structures.

**Figure 2:**
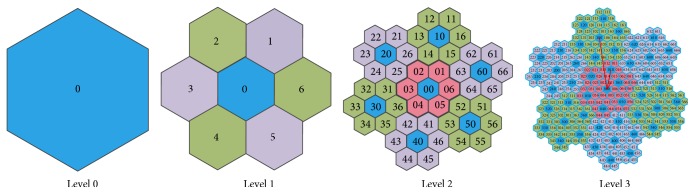
Honeycomb structure on different level.

Honeycomb structure has obvious self-similar fractal characteristics. We show the honeycomb structure as in Figure 2.

**Figure 3:**
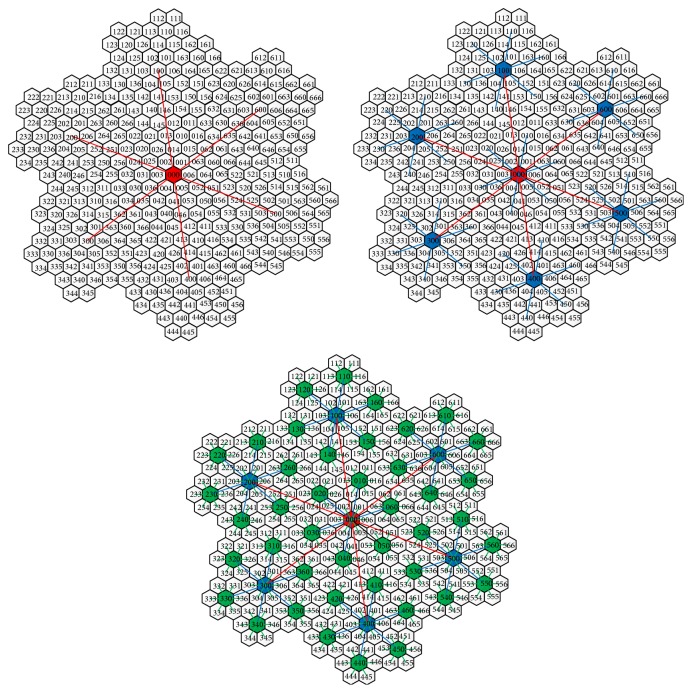
Honeycomb structure management method.

For any two-dimensional space, we can get the complete coverage for it by different level of honeycomb structure with proper radius of hexagon cell. The honeycomb structure can be divided into 7 blocks. We give each block an ID: the central block is 0 and six surrounding blocks are 1 to 6, respectively; with fractal method, we can give each cell an ID, as shown in Figure 2. With the cell ID, we can easily find the exact block, sub-block and the cell position at the whole space. The hierarchic management of honeycomb is shown in Figure 3.

Each honeycomb structure can be divided into seven substructures; each substructure is also a honeycomb structure. So the whole structure is a self-similar hierarchic structure. We think it is also very useful to manage many real-world problems efficiently. The recursion structure of honeycomb (in C# format) is as in Algorithm 1.

Suppose the central cell is *C*_0_, the edge length of cell is *α*, and the level index is *n*; there are some useful equations for our algorithm. Equation (9) represents the area that the honeycomb structure can cover:

(9) An=7nA0,A0=α2·332.

Equation (10) represents the distance between subblock center and upper-level center:

(10) $$\begin{array}{c} ƛ=\sqrt{3\cdot 7^{n-1}}\cdot \alpha \;n\geq 1. \end{array}$$

So the distance between cell ID* 100* and cell ID* 000* in Figure 1 (Level 3) is $\sqrt{3\cdot 7^{3-1}}\cdot \alpha$.

Equation (11) represents the rotation angle of subblock center to upper-level center:

(11) $$\begin{array}{c} \omega =\frac{\iota \cdot \pi }{3.0}+\arctan\left(\;\frac{\sqrt{3}}{5}\;\right)\cdot \left(\;n-1\;\right), \end{array}$$

where *ι* ∈ [1,6] is the subblock index. So the rotation angle of center of cell ID* 100* is $1\cdot \pi /3.0+arctan(\sqrt{3}/5)\cdot (3-1)$.

Supposing the minimum distance of any two points (including depot and demand points) in this space is *d*_*ij*_^*∗*^, if *d*_*ij*_^*∗*^ > 2*α*, then there is at most one point in a cell. After some experiments, we suggest that the level number should be less than 6, because when *n* ≥ 7, the honeycomb construction process is time consuming to personal computer; when *n* = 6 the total cells are 7^6^ which is enough for ordinary VRP. For some extreme example, we could allow more than one command point in one cell; when the initial solution of TSP is generated, we could give a random sequence of the points in one cell.

According to the characteristic of the honeycomb structure, we use the recursion procedure to generate a honeycomb structure. The pseudocode of the algorithm in C# format is shown in Algorithm 2.

By a similar recursion method, we also can give every cell an ID; according to the management principle, all the cells in honeycomb structure can form a sequence. If the cell contains demand points, we push the point into a stack in order; then we can also get the order of demand point; this order of demand point is the initial solution of the TSP problem. Based on this initial problem, we can calculate the final solution of VRP by adding the constraints.

#### 3.2.2. Improvement by Genetic Algorithm (GA)

Based on the initial solution calculated by honeycomb based SFC, we use GA to improve the solution. GA is a widely used artificial intelligent method; there are many papers about the details of the algorithm; we only introduce the modifications of our method to fit the VRP.

*(1) Chromosome Representation.* In this paper, we use the classic chromosome representation to VRP, using a string to be the chromosome, but the minimum unit of string is the numeric number of demand points; 0 represents the depot.

*(2) Initial Population Generation.* Classic population is randomly generated. There are some other improved population generation methods for VRP, such as the method based on the sweep approach of Gillett and Miller [14] or solutions based loosely on the generalized assignment approach of Fisher et al. [15]. Due to the characteristics of honeycomb structure, the initial solution has a certain degree of improvement; we use Or-opt method to generate an initial population of GA to search the optimal solution of VRP.

**Figure 4:**
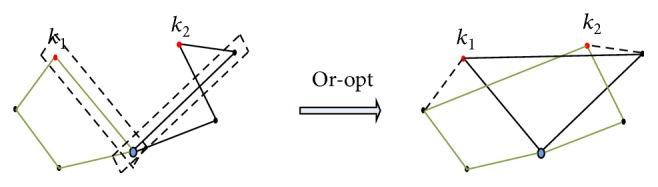
2-opt method for VRP.

Taking the 2-opt method into consideration to illustrate the process of algorithm, in Figure 4, which is a solution of simple VRP, there are two vehicles to finish the distribution task; we choose point *k*_1_ in first vehicle's route and point *k*_2_ in second vehicle's route and then exchange the position of points *k*_1_ and *k*_2_ in each other's vehicle route; then we get a new solution. After the completion of or-opt process, we choose the optimal solution of the population and always keep the chromosome of the optimal solution directly to the next generation, this operation can improve the rate of convergence of the algorithm.

Suppose the local search step length for Or-opt is *s*_*l*_; usually *s*_*l*_ ≤ 4. The best solution of initial population is *R*_opt_^*∗*^. The algorithm to generate the initial population of GA is shown in Algorithm 3.

**Figure 5:**
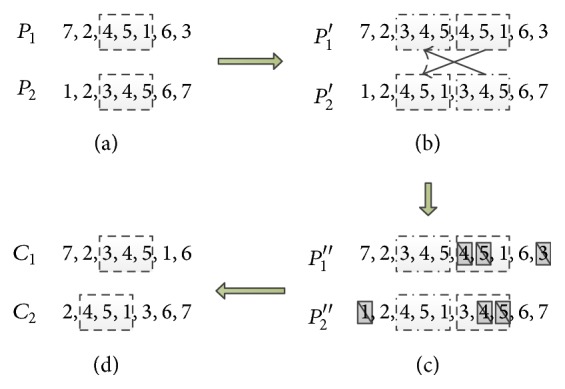
Crossover operation.

*(3) Crossover Operator.* Crossover operator is a major process of producing children solutions from current population. There are many methods for crossover operation according to different problems. We introduce an easy crossover operator to fulfill the task. The crossover operation for a simple example with 7 demand points is shown in Figure 5.

The process is described as follows.

**Step 1 .**

Randomly choose two chromosomes as parents and then generate crossover chromosome segment randomly. Generate two integers randomly: one for crossover point and the other for segment length, as shown in Figure 5(a).

**Step 2 .**

Swap the crossover genes segment, as shown in Figure 5(b).

**Step 3 .**

Validity checking: due to the constraints of VRP, each demanding point can only be visited once; keep the crossover gene segment; delete the same number in the parent chromosomes, as shown in Figure 5(c).

**Step 4 .**

Get two new chromosomes with crossover gene segment and save them to the next generation, as shown in Figure 5(d).

#### 3.2.3. Interpolation Operator

Interpolation operator is a process to convert solution of TSP to solution of VRP. The interpolation operator is to put depots into the sequence of demand points; each segment separated by depot represents one vehicle's delivery task. Take the chromosome *C*_2_ in Figure 5(d) as an example. (2, 4, 5, 1, 3, 6, 7) is one solution of seven-point TSP. Insert point “0” into the solution, such as (0, 2, 0, 4, 5, 0, 1, 3, 6, 7, 0), which becomes a solution of seven-point one-depot VRP. The solution means there are three vehicles to finish the distribution work: the first segment “0-2-0” represents that the first vehicle delivers goods for demand point 2 and then back to depot; the second segment “0-4-5-0” represents the second vehicle's route and task. Where to insert depot point depends on vehicle capacity and time windows constraints.

The process of interpolation operator is the iteration of checking the sequence of demanding point one by one, accumulates the demand volume, arrival time to the next point; once they cannot satisfy the constraints, insert “0” point behind the point and then begin a new iteration.

### 3.3. Time Complexity Analysis of the Algorithms

According to the previous part, we analyze the time complexity of the algorithm step by step as follows.

*(1) Construction of the Honeycomb.* Using the recursion procedure to construct the honeycomb structure and give the ID to every cell, the time complexity of the procedure is *O*(7^*n*^) where *n* is the level number of the honeycomb. As we mentioned before, we suggest that *n* < 7 to get an initial solution fast.

*(2) Get the Initial Solution of VRP.* Get the initial solution of VRP by sorting the cell ID which contains more than one demand point. The time complexity of sorting process is *O*(*l* · log*l*), where *l* is the demand points number.

*(3) Or-opt Method to Generate the Population of GA.* The time complexity of Or-opt algorithm is *O*(*l* ^*r*^), where *l* is the demand points number and *r* is the exchange segment length. Take the 2-opt method as an example; the time complexity is *O*(*l* ^2^).

*(4) Genetic Algorithm to Improve the Solution.* According to the process of GA, we should execute three operations at each iteration: selection, crossover operator, and mutation operator.

The time complexity of selection is *O*(*ω*), where *ω* is the population size, the time complexity of mutation operator is *O*(*ω* · *ρ*), where *ρ* is the mutation segment length, the time complexity of crossover operator is *O*(*ω* · *l*). Therefore, the time complexity of every iteration is *O*(*ω* · *l*), the total time complexity is *O*(*ω* · *l* · *I*), where *I* is the maximum iteration number of GA. Generally iteration number is bigger than demand point number, *I* > *l*, so the time complexity in this step is *O*(*l* ^3^).

Based on the above analysis, the time complexity of the HOA is *O*(*l* ^3^), determined by GA.

## 4. Solomon Experimentation

We implemented experiment software by C# on Windows 7 OS and conducted a lot of computation experiments on famous Solomon's benchmark. Solomon's benchmarks have 56 instances and include 25 customers serial instances, 50 customers serial instances, and 100 customers serial instances. These 56 problems are categorized into six classes, namely, C1, C2, R1, R2, RC1, and RC2. Problems which fall into C categories are clustered data, meaning nodes are clustered either geographically or in terms of time windows. Problems from R categories are uniformly distributed data and those from RC categories are hybrid problems that have the features of both C and R categories. In addition, C1, R1, and RC1 problem sets have narrow time window for the depot, whereas C2, R2, and RC2 have wider time window for the depot. The best solution published so far for Solomon's benchmarks can be found through the following link: http://www.sintef.no/Projectweb/TOP/VRPTW/Solomon-benchmark/.

The computation experiments are performed on a personal computer with Intel Core 2 Duo 2.6 G CPU and 4 G RAM; the parameters for the algorithm are defined as follows: maximum honeycomb level is 6, VRP is with hard time window, the two objective functions use the hierarchy of minimizing number of vehicles rather than minimizing total distance, 2-opt method is used to generate the initial population of GA, the population size is 500, crossover rate is 40%, the mutation rate is 10%, and the iteration number is 500. The computation results are shown in Table 1.

For 25 demand points' group, the average deviation to the best known solution is 8.92%. However, the average deviations for 50 demand points' group and 100 demand points' group are 15.39% and 19.32%, respectively. The results show that this HOA can efficiently solve small scale problems. Specifically, HOA can find the optimal solution in a very short CPU time for 25 demand points' group, which is highlighted in Table 1.

With regard to large scale problems (100 demand points' benchmarks), the CPU time and best solutions of different algorithms are compared as follows. Note that the computation results of 2-INT, SA, Tabu, and GA can be found at [2].

**Figure 6:**
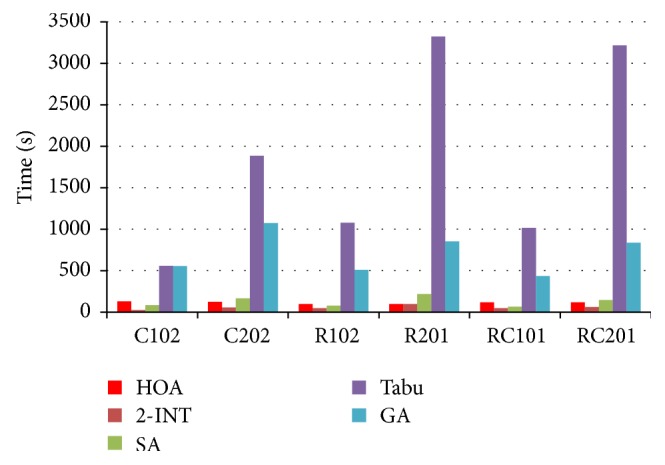
CPU time comparison (s).

**Figure 7:**
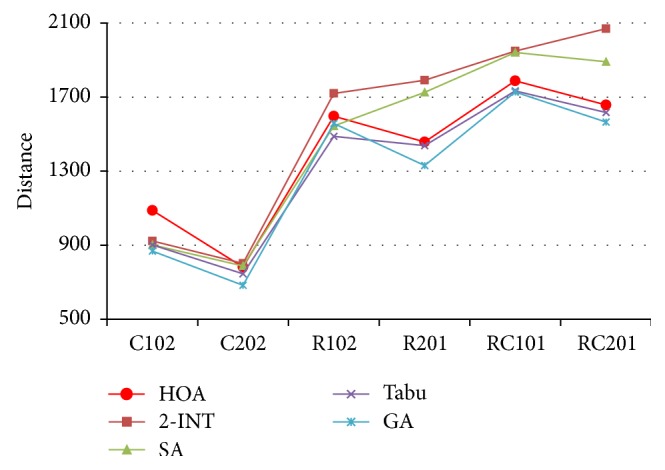
Results comparison.

The compared results are shown in Table 2, and the results are also presented in Figures 6 and 7.

According to Figures 6 and 7, we can easily find that the HOA can obtain promising computation efficiency.

Compared to 2-INT, although the computation cost is slightly worse, the solutions obtained by the HOA are much better for most instances (e.g., types R1, R2, RC1, and RC2).

Compared to SA, the computation cost is very similar, but the HOA produces better solutions for most instances (e.g., types R2, RC1, and RC2).

Compared to Tabu, the HOA can obtain very similar solutions by amazing computation time, less than 1/10 the computation time of Tabu.

Compared to GA, the HOA produces similar solutions in much less computation time. Note that a similar solution to classic GA can be produced by HOA, if we adjust algorithm parameters (such as population size and iteration number) of HOA properly.

The computation costs by using HOA for all instances are very close; the difference between the fastest instance (R102, R201) and the slowest instance (C102) is only 33 s.

**Figure 8:**
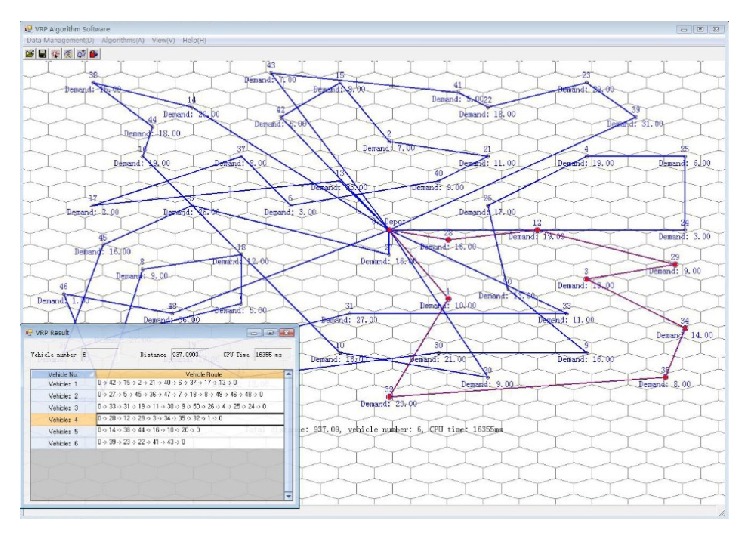
Results of R201 graph.

To summarize, we can conclude that the HOA has better computation efficiency and robustness to VRPs and has a real potential application for its fast computation efficiency and robustness. It can provide a feasible solution according to customer's requirement; for example, customer can customize the iteration number before the algorithm begins; even in a few iterations it can output an improved solution. To deal with small or medium scale problems, HOA can produce a good or even the optimal solution in very short time.  Figure 8 is the experimental software interface to solve the 50 demand points of R201; it can find the near-optimal solution within 20 seconds. For 25 demand points' problem, it can find the solution within a few seconds.

## 5. Conclusions

This paper proposes a fractal hierarchic honeycomb structure based HOA to solve VRP. The algorithm first divides the whole space into sequenced cells by honeycomb structure to get the initial solution of VRP, uses GA to improve an initial solution, and gets the final solution for VRP. Experimental software is developed based on the proposed HOA, and computational experiments are conducted based on Solomon benchmark. The test results demonstrate the superiority of the proposed algorithm.

This is the first time to implement the honeycomb structure and apply it to VRP; the self-similar hierarchic honeycomb structure has many characteristics, and we provide a new cell ID sequence method based on fractal theory. The hierarchic management idea of honeycomb structure can potentially be applied into different real-world management problems, such as large scale social management and grid management.

The proposed HOA has been proved to be computationally efficient and robust for VRP. Moreover, it can be easily implemented and potentially can be generalized to solve various real work problems.

## Figures and Tables

**Algorithm 1:**
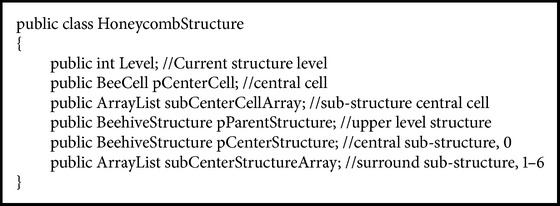
Definition of honeycomb structure.

**Algorithm 2:**
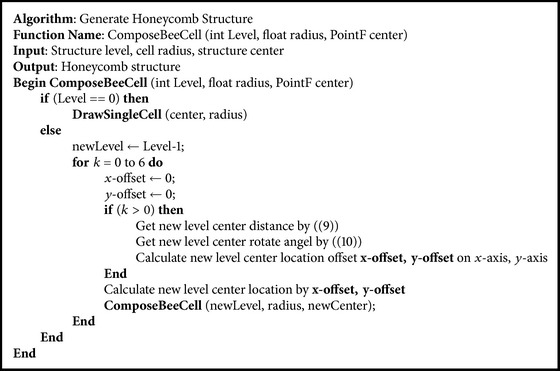
Honeycomb structure generating algorithm.

**Algorithm 3:**
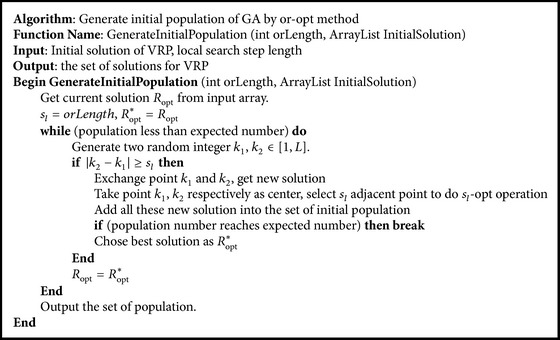
Or-opt method to generate the initial population.

**Table 1:** Comparison of results for Solomon's benchmark.

Type	Results (distance/vehicle number)			CPU time (ms)			Best known solution (distance/vehicle number)			Gap Δ (%)		
	25	50	100	25	50	100	25	50	100	25	50	100
	points	points	points	points	points	points	points	points	points	points	points	points
C101	**191.8/3**	434.35/7	1094.83/13	7843	20437	130656	191.3/3	362.4/5	828.94/10	0	19.8	32.1
C102	**196.08/3**	398.39/6	1087.73/12	7640	19968	129375	190.3/3	361.4/5	828.94/10	3.0	10.2	31.2
C201	**219.23/2**	434.96/4	849.64/5	7281	17656	123796	214.7/2	360.2/3	591.56/3	2.1	20.7	43.6
C202	241.46/2	422.91/3	782.3/5	7203	17218	124500	214.7/2	360.2/3	591.56/3	12.5	17.4	32.2
R101	654.71/8	1108.65/14	1806.0/22	8656	21890	97859	617.1/8	1047.0/12	1645.79/19	6.1	5.9	9.7
R102	580.06/7	999.24/12	1596.55/18	8125	20734	95953	547.1/7	944.9/12	1486.12/17	6.0	5.7	7.4
R201	**514.85/3**	951.98/6	1458.06/8	6843	21355	95921	463.3/4	800.7/6	1252.37/4	11.1	18.9	16.4
R202	**486.85/3**	846.61/5	1331.56/5	6750	17687	95406	410.5/4	712.2/5	1181.70	18.6	18.9	12.7
RC101	490.0/5	1014.41/10	1788.93/16	7828	20984	115890	461.1/4	957.9/9	1696.94/14	6.3	5.9	5.4
RC102	**351.9/3**	999.68/9	1694.35/15	7625	20531	114921	351.8/3	844.3/8	1554.75/12	0	18.4	9.0
RC201	427.29/3	888.31/6	1658.15/6	7843	17937	116640	360.2/3	684.8/5	1406.91/4	18.6	29.7	17.9
RC202	414.97/3	694.9/4	1558.91/6	6562	17828	118406	338.0/3	613.6/5	1365.65/3	22.8	13.2	14.2

**Table 2:** Comparison of our results with the historical best (time(s)/distance).

Type	Algorithm				
	HOA	2-INT	SA	Tabu	GA
C102	129/1087.73	25/923.38	84/901.53	557/901.53	556/868.80
C202	124/782.3	55/801.28	166/787.86	1885/746	1073/683.86
R102	96/1596.55	46/1720.46	78/1544.82	1076/1488.59	507/1558.59
R201	96/1458.06	98/1791.42	217/1726.13	3323/1437.49	851/1329.74
RC101	116/1788.93	49/1948.94	63/1940.57	1016/1734.17	432/1728.3
RC201	116/1658.15	60/2070.4	146/1891.9	3217/1617.5	835/1565.67
